# Correlation of Iron Deficiency Anaemia With Depression Severity: Evidence From an Observational Study in Rural India

**DOI:** 10.7759/cureus.96391

**Published:** 2025-11-08

**Authors:** Sesha Deepthi, Mohammed Abdul Salaam, Prasanna Kumar Kancharlapalli, Yadavalli RD Rajan

**Affiliations:** 1 Pathology, Konaseema Institute of Medical Sciences, Amalapuram, IND; 2 Psychiatry, Konaseema Institute of Medical Sciences, Amalapuram, IND; 3 Psychiatry, Mamata Medical College, Khammam, IND; 4 Plastic and Reconstructive Surgery, Sri Venkateswara Medical College, Tirupati, IND

**Keywords:** depression, iron deficiency and depression, iron deficiency anemia (ida), nutritional deficiency, suicide prevention

## Abstract

Introduction

Depression is a common mental health condition that can occur alongside other physical or nutritional problems. This study examines the relationship between iron deficiency and the severity of depressive symptoms.

Aims

To investigate the correlation between iron deficiency anaemia (IDA) and depression severity and to assess the influence of socio-demographic factors.

Methods and materials

A cross-sectional study was conducted on 200 patients aged 16-60 years with depression (ICD-11) and confirmed IDA (haemoglobin <13 g/dl in males, <12 g/dl in females, mean corpuscular volume (MCV) <80 fL). Depression severity was assessed using the Montgomery-Åsberg Depression Rating Scale (MADRS). Sociodemographic data, complete blood counts, and peripheral smears were obtained. Data analysis included descriptive statistics, Spearman’s correlation, and multivariable regression. Data were analysed using R (v4.0.2; R Foundation for Statistical Computing, Vienna, Austria) with descriptive statistics, the Shapiro-Wilk test for normality, and Spearman’s correlation. A p-value <0.05 was considered statistically significant.

Results

Participants had a mean age of 40.1 ± 4.2 years; females comprised 64.5% (n=129), and 81% (n=162) resided in rural areas. Severe depression (MADRS >34) was observed in 55.5% (n=111) of participants. Most blood smears (n=141; 70.5%) showed a microcytic hypochromic pattern. Significant negative correlations were found between MADRS scores and haemoglobin (rho = -0.45, p <0.001), MCV (rho = -0.45, p <0.001), mean corpuscular haemoglobin (MCH) (rho = -0.31, p <0.001), and mean corpuscular haemoglobin concentration (MCHC) (rho = -0.34, p <0.001). Regression analysis confirmed haemoglobin as an independent predictor of depression severity (β = -1.58, p <0.001).

Conclusion

IDA is significantly associated with greater depression severity in rural Indian patients. Screening and addressing anaemia alongside antidepressant therapy may provide a cost-effective strategy to improve mood outcomes, highlighting the need for integrated management approaches.

## Introduction

Depression is one of the most prevalent mental health disorders globally, affecting approximately 5% of adults and contributing significantly to years lived with disability [1]. It manifests as a persistent disturbance in mood, cognition, and motivation, and is associated with increased risk of chronic illnesses such as diabetes, hypothyroidism, and cardiovascular disease, as well as higher mortality from suicide [2,3]. Beyond its psychological symptoms, depression has strong biological underpinnings involving inflammation, neurotransmitter dysregulation, and alterations in brain circuitry [3-5]. These shared biological pathways explain why depression frequently coexists with systemic medical conditions, amplifying their impact on functional well-being and overall morbidity [2].

Among nutritional and metabolic contributors, iron deficiency anaemia (IDA) remains one of the most widespread global health problems, affecting nearly a quarter of the world’s population and showing disproportionately high prevalence in low- and middle-income countries such as India [6]. Women of reproductive age, adolescents, and rural communities are particularly affected due to nutritional inadequacies and physiological losses. Iron is an essential micronutrient for oxygen transport and neuronal metabolism; it also plays a crucial role in the synthesis of monoamine neurotransmitters such as serotonin, dopamine, and norepinephrine. Deficiency of iron can impair neurotransmitter synthesis, myelination, and synaptic plasticity, leading to altered mood regulation, cognitive decline, and behavioural changes [5,7].

Emerging research has revealed a possible bidirectional relationship between iron deficiency and depressive disorders. Several case-control and population-based studies have demonstrated that individuals with anaemia have higher depression scores and prevalence of depressive symptoms compared to those with normal haemoglobin (Hb) levels [8-11]. Biological evidence suggests that reduced iron availability impairs enzymatic pathways responsible for monoamine synthesis, leading to imbalances in serotonin and dopamine activity [5,7]. Furthermore, iron deficiency is associated with heightened neuroinflammation and oxidative stress, with elevated levels of cytokines such as IL-1β, IL-6, and TNF-α contributing to depressive pathophysiology [3-5]. Given India’s high burden of IDA and rising rates of depression, it is critical to understand how these two prevalent conditions interact. Despite growing international literature, data from Indian populations remain sparse.

The present study was therefore undertaken to investigate the correlation between iron deficiency anaemia and the severity of depression in an Indian cohort. Understanding this relationship could provide insights into early screening, improve integrated management of depression, and highlight anaemia as a potentially modifiable biological risk factor for mood disorders.

## Materials and methods

This cross-sectional study was conducted over two years, from 2023 January to 2025 January, in the Department of Psychiatry, Amalapuram, Andhra Pradesh, to study the association between IDA and the severity of depression. The sample size was calculated using Fisher’s z transformation for correlation studies, assuming a two-sided α = 0.05, power = 80%, and an expected correlation of r = 0.20 between Hb and Montgomery-Åsberg Depression Rating Scale (MADRS) scores. The required sample size was 194, which was increased to 200 to account for potential non-response and missing data. A total of 200 consecutive OPD patients aged 16-60 years, diagnosed with mild to severe depression as per International Classification of Diseases 11th Revision (ICD-11) and confirmed to have iron deficiency anaemia (Hb <13 g/dl in males, <12 g/dl in females, mean corpuscular volume (MCV) <80 fL with microcytic hypochromic smear), were included in the study. Patients with other anaemias, psychiatric disorders, severe medical illness, recent alcohol use, pregnancy, or on iron supplementation were excluded from the study. After obtaining informed consent, socio-demographic details such as age, gender, domicile, education, occupation, marital status, income, and religion were recorded using a structured proforma. Each participant underwent a comprehensive clinical and laboratory evaluation, including haemoglobin estimation, MCV, mean corpuscular haemoglobin (MCH), mean corpuscular haemoglobin concentration (MCHC), and peripheral blood smear examination to confirm IDA. The severity of depression was assessed using the MADRS [12], a clinician-administered scale consisting of 10 items measuring the intensity of depressive symptoms. The obtained results were analyzed using the R programming language (version 4.0.2, R Foundation for Statistical Computing, Vienna, Austria) with RStudio (Posit PBC, Boston, MA, USA) as the integrated development environment. The analysis included computation of means, standard deviations, and percentages according to the study objectives. Categorical variables were summarized as frequencies and percentages, while quantitative variables were expressed as means and standard deviations. The Shapiro-Wilk test was used to assess data normality, and Spearman’s rank correlation test evaluated relationships between variables. Scatter plots were generated to visualize correlations, and diagrammatic representations were used to depict findings. A p-value of less than 0.05 was considered statistically significant.

## Results

The study included 200 participants aged 18-60 years, with the mean age being 40.1 ± 4.2 years. The largest subgroup (n=62; 31%) was between 29-38 years, followed by 27.5% (n=55) in the 49-60 age range. Females constituted nearly two-thirds of the sample (n=129; 64.5%), and the majority of participants resided in rural areas (162; 81%). In terms of education, most had studied up to secondary school (n=120; 60%), while 23% (n=46) were graduates. Occupation-wise, housewives accounted for half of the sample, followed by employed individuals (n=54; 28%) and unemployed participants (n=44; 22%). A large proportion were married (n=173; 86.5%), and Hindus formed the majority religion (n=150; 75%). The socio-demographic profile of the study population is shown in Table 1. The MADRS scores of the sample are described in Table 2. More than half of the patients (55.5%; n=111) with depression show MADRS score >34, indicating severe depression, followed by moderate depression with MADRS score 20-34 (26%; n=52).

**Table 1:** Socio-demographic profile of the study population

Variable	Category	Frequency (n)	Percentage (%)
Age (years)	16–28	32	15.9
	29–38	62	31.0
	39–48	52	26.0
	49–60	54	27.5
Gender	Male	71	35.5
	Female	129	64.5
Domicile	Rural	162	81.0
	Urban	38	19.0
Education	Illiterate	4	2.0
	Primary school	29	14.5
	Secondary school	121	60.5
	Graduate	46	23.0
Occupation	Housewife	99	49.5
	Unemployed	44	22.0
	Employed	57	28.5
Monthly Income (INR)	1,000 – 5,000	95	48.0
	5,100 – 10,000	90	43.0
	> 10,000	15	9.0
Religion	Hindu	150	75.0
	Christian	27	13.5
	Muslim	23	11.5
Marital Status	Married	173	86.5
	Unmarried	27	13.5

**Table 2:** MADRS scores distribution of sample MADRS- Montgomery-Åsberg Depression Rating Scale

MADRS score	Frequency	Percent	Valid percent	Cumulative percent
0-6	0	0	0	0
7-19	37	18.5	18.5	18.5
20-34	52	26	26	44.5
>34	111	55.5	55.5	100
Total	200	100	100	100

Among the 200 participants, the majority (n=141; 70.5%) showed a microcytic hypochromic blood picture. A smaller proportion (n=7; 3.5%) demonstrated macrocytic hypochromic anaemia, while 24 participants (12%) were found to have normocytic normochromic anaemia. The mean values of haemoglobin indices are described in Table 3 and the correlation between MADRS and haemoglobin indices is described in Table 4.

**Table 3:** Mean values of haemoglobin indices and MADRS MADRS- Montgomery-Åsberg Depression Rating Scale; MCV- Mean Corpuscular Volume; MCHC- Mean Corpuscular Haemoglobin Concentration; MCH- Mean Corpuscular Haemoglobin

Variables	Mean ∓ Standard deviation	W-statistic (Shapiro-Wilk test)	p-value
Haemoglobin	9.7 ∓ 2.4	0.981	0.009
MCV	74.2 ∓ 11.7	0.884	<0.001
MCHC	27.1 ∓ 4.1	0.985	0.036
MCH	24.4 ∓ 3.8	0.980	0.006

**Table 4:** Correlation between MADRS and clinical variables MADRS- Montgomery-Åsberg Depression Rating Scale; MCV- Mean Corpuscular Volume; MCHC- Mean Corpuscular Haemoglobin Concentration; MCH- Mean Corpuscular Haemoglobin Spearman’s rank correlation, p-value <0.05 is considered significant

Variables	p-value	rho-value
Haemoglobin	<0.001	-0.4465091
MCV	<0.001	-0.4487246
MCH	<0.001	-0.3143924
MCHC	<0.001	-0.3350049

**Figure 1:**
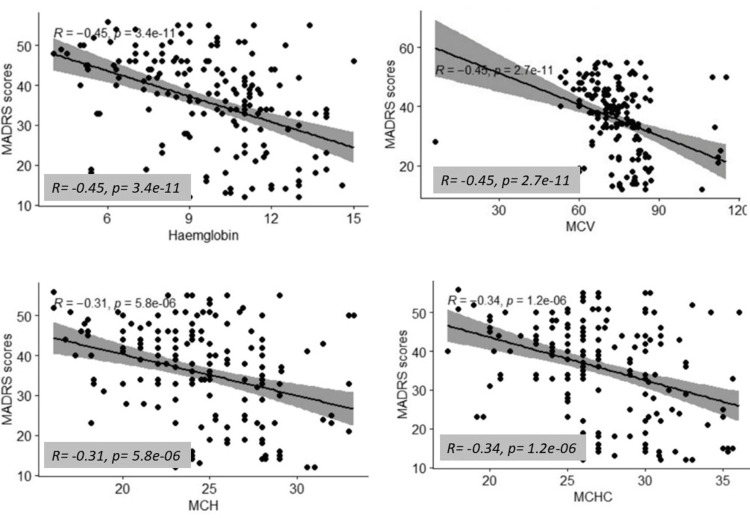
Correlation between haemoglobin indices and MADRS MADRS- Montgomery-Åsberg Depression Rating Scale

The scatterplot analyses (Figure 1) demonstrated significant negative correlations between haematological indices and depression severity as measured by MADRS scores. Hb showed a moderate negative correlation (rho = -0.45, p < 0.001), indicating that lower Hb levels were consistently associated with higher MADRS scores and more severe depression. Similarly, MCV displayed a negative correlation with depression severity (rho = -0.45, p < 0.001), suggesting that reduced red cell size was linked to increased depressive symptom burden. MCH also correlated negatively with MADRS scores (rho = -0.31, p < 0.001), showing that lower MCH was associated with higher depression severity. In addition, MCHC revealed a significant negative correlation (rho = -0.34, p < 0.001), further supporting the observation that lower red cell indices are linked with worsening depression.

A multivariable linear regression analysis (*R^2^*=0.297*)* was performed to predict the MADRS scores. Normality of residuals showed an approximately normal distribution (p > 0.05), and variance was homoscedastic across fitted values. Thus, the assumptions for linear regression were considered met. Multicollinearity was evaluated using Variance Inflation Factors (VIFs), which revealed high collinearity among Hb, MCV, MCH, and MCHC. Hb was found to be a significant independent predictor, with lower Hb levels associated with higher depression severity (β = -1.58, 95% CI -2.34 to -0.83, p = 0.0001). MCHC showed a borderline association (β = -0.82, 95% CI -1.64 to 0.00, p = 0.050), while MCV and MCH were not significant predictors. Among demographic factors, male gender (β = -6.15, p = 0.024) and urban domicile (β = -4.35, p = 0.040) were significantly associated with lower MADRS scores compared to female and rural participants (Table 5).

**Table 5:** Multivariable Linear Regression Comparing Haemoglobin indices and demographic factors to MADRS Scores MCV- Mean Corpuscular Volume; MCHC- Mean Corpuscular Haemoglobin Concentration; MCH-Mean Corpuscular Haemoglobin

Predictor	β (Coefficient)	95% CI	p-value
Haemoglobin (Hb)	–1.58	–2.34 to –0.83	0.0001
MCV	–0.08	–0.26 to 0.09	0.346
MCH	0.29	–0.61 to 1.18	0.524
MCHC	–0.82	–1.64 to 0.00	0.050
Male	–6.15	–11.48 to –0.83	0.024
Urban	–4.35	–8.50 to –0.21	0.040

## Discussion

The present study investigated the association between IDA and the severity of depression in a rural Indian population. Our findings demonstrate a significant negative correlation between haematological indices (Hb, MCV, MCH, MCHC) and MADRS scores, indicating that individuals with lower red cell parameters had more severe depressive symptoms. More than half of the participants were classified as having severe depression, and the majority of blood smears (70.5%) revealed a microcytic hypochromic picture, confirming IDA as the dominant haematological abnormality. These results provide supportive evidence that iron deficiency may play a crucial role in modulating the clinical expression of depression.

Our findings align with several studies worldwide that highlight the close link between iron deficiency and mood disorders. Shafi et al. [8] demonstrated significantly lower Hb levels among depressed individuals compared to controls, with a negative correlation coefficient of -0.429, which is comparable to the rho values observed in our cohort. Similarly, Hidese et al. [9] reported that women with self-reported IDA exhibited substantially higher psychological distress. Noorazar et al. [10] and Alkhalidi et al. [11] also emphasised the relationship between iron deficiency anaemia and depressive disorders in clinical populations. Meta-analyses, such as that by Azami et al. [13], further confirm that anaemia, particularly postpartum anaemia, increases the risk of depression. Taken together, these reports suggest that anaemia can be considered as one of the potentially modifiable risk factors for depression.

The biological mechanisms underlying this association are multifactorial. Iron plays a pivotal role in the synthesis and regulation of monoamine neurotransmitters, particularly serotonin, dopamine, and norepinephrine. Reduced iron availability impairs the activity of aromatic amino acid hydroxylases, the rate-limiting enzymes in neurotransmitter synthesis, leading to neurotransmitter imbalance and impaired mood regulation. Beyond neurotransmission, iron is essential for myelination and synaptogenesis, and deficiency during adulthood can impair neuronal connectivity, particularly in regions such as the prefrontal cortex, amygdala, and hippocampus, which are central to emotional regulation. Furthermore, IDA is associated with increased neuroinflammation, with elevated cytokines (IL-1β, IL-6, TNF-α) contributing to reduced serotonin bioavailability and worsening depressive pathology [3-5]. These overlapping pathways explain the consistent observation of higher depression severity with lower iron indices in our study.

Another important observation in our study is the lack of significant association between socio-demographic variables and depression severity. Previous research has highlighted gender differences, with women often reporting a higher prevalence of both anaemia and depression. For instance, Onder et al. [14] reported that anaemia was disproportionately linked with depressive symptoms in women, largely due to nutritional deficiencies and physiological factors such as menstruation and pregnancy. Similarly, rural residency and lower education levels have been reported to increase vulnerability to depression through socioeconomic hardship and nutritional inadequacy [15,16]. The absence of such associations in our study may be attributable to the homogeneity of our sample, as most participants were rural, married women with modest education levels. This highlights that while socio-demographic differences may influence prevalence across populations, biological factors like iron deficiency may play a more consistent role in determining depression severity.

Our results also resonate with the clinical observation that anaemia and depression share overlapping symptomatology, including fatigue, poor concentration, irritability, and low motivation. These overlapping features can complicate diagnosis, as anaemia-induced fatigue may be misattributed to depression or vice versa. Moreover, chronic anaemia reduces oxygen delivery to the brain, impairing cognitive performance and emotional regulation [17,18]. This dual burden may exacerbate functional impairment in patients, making recognition and treatment of anaemia crucial in comprehensive mental health care. A few reports have stated that correction of anaemia, particularly with iron supplementation, improves mood symptoms and cognitive performance, suggesting that addressing anaemia may directly improve depressive outcomes [7].

Finally, our study highlights important public health implications. India continues to face a high burden of IDA, particularly among women of reproductive age, adolescents, and rural populations. At the same time, depression remains a leading cause of disability-adjusted life years (DALYs). The coexistence of these conditions in vulnerable populations may create a vicious cycle where nutritional deficiencies worsen depression, and depression in turn reduces appetite, dietary intake, and compliance with supplementation. Screening for anaemia in depressed patients and for depressive symptoms in anaemic individuals may therefore represent a cost-effective strategy for early detection and integrated management. Future research should incorporate larger, multicentric cohorts and biochemical markers such as serum ferritin, transferrin saturation, and soluble transferrin receptor to strengthen diagnostic precision. Interventional studies assessing the effect of iron supplementation, alongside antidepressants, on depression outcomes are also warranted.

This study has certain limitations, including its single-centre design, relatively small sample size, and predominance of rural participants, which may restrict the generalisability of the findings. The diagnosis of iron deficiency anaemia was based primarily on haemoglobin and red cell indices, without the inclusion of advanced markers such as serum ferritin, transferrin saturation, or soluble transferrin receptor, which could have provided greater diagnostic accuracy, and the cross-sectional nature of the study prevents establishing causality. Confounding factors like antidepressant usage and chronic co-morbid illness have not been eliminated. Also, this study was done on patients with both IDA and depression, thus reducing the generalisability for a larger population. Future research should adopt larger, multicentric cohorts, incorporate comprehensive biochemical markers, and include longitudinal or interventional designs to evaluate whether correction of iron deficiency with supplementation can directly improve depressive outcomes.

## Conclusions

This study demonstrates a significant inverse relationship between iron deficiency anaemia and depression severity, with lower haemoglobin and red cell indices correlating with higher MADRS scores. These findings underline the importance of routine screening and integrated management of IDA in patients with depression. Addressing IDA through timely diagnosis and supplementation, alongside standard antidepressant therapy, may serve as a cost-effective strategy to improve mood outcomes and quality of life, particularly in vulnerable populations.
